# Antioxidant Contributors in Seed, Seed Coat, and Cotyledon of γ-ray-Induced Soybean Mutant Lines with Different Seed Coat Colors

**DOI:** 10.3390/antiox10030353

**Published:** 2021-02-26

**Authors:** You Jin Lim, Soon-Jae Kwon, Shanshan Qu, Dong-Gun Kim, Seok Hyun Eom

**Affiliations:** 1 Department of Horticultural Biotechnology, Institute of Life Sciences & Resources, Kyung Hee University, Yongin 17104, Korea; yujn0213@khu.ac.kr (Y.J.L.); a1352185649@sina.com (S.Q.); 2 Advanced Radiation Technology Institute, Korea Atomic Energy Research Institute, Jeongeup 56212, Korea; soonjaekwon@kaeri.re.kr (S.-J.K.); 1130029@scnu.ac.kr (D.-G.K.)

**Keywords:** soybean, gamma-ray, antioxidant activity, anthocyanin, epicatechin, isoflavone

## Abstract

The development of soybean with high antioxidant activities for use in the food and cosmetics industries is a target of breeding programs. In soybean, antioxidants are associated with seed color, although the metabolic basis for seed coloration remains incompletely understood. We selected six γ-ray-induced mutant lines that exhibited black, partially black, brown, partially brown, or yellowish-white pigmentation in the seed coat. Antioxidant activity and contents of anthocyanins, flavan-3-ols, and isoflavones were evaluated in the seed coat and cotyledons. The lines with black or brown seeds showed the highest antioxidant activities. The cotyledons showed no significant differences in seed coat components or antioxidant activities among lines. Black and brown seed coat components showed the highest antioxidant activities. The black seed coat contained five anthocyanins, whereas seed coats of brown- and yellow-seeded lines entirely lacked anthocyanins. Both black and brown seeds were rich in flavan-3-ols, including catechin and epicatechin, which were the predominant antioxidant contributors in brown seeds. Isoflavone contents showed weaker correlations with antioxidant activity than anthocyanins and flavan-3-ols. These results demonstrated that antioxidant activities were determined by anthocyanins in black seeds and flavan-3-ols in brown and black seeds, whereas relatively low antioxidant activities in yellow seeds reflected their high isoflavone contents.

## 1. Introduction

Soybean (*Glycine max* (L.) Merr.) is a nutritionally rich and well-balanced crop utilized for a variety of applications. Soybean is a valuable industrial material used for health supplements, cosmetics, and pharmaceutical products. To develop cultivars with enhanced functional properties, soybean breeding has employed diverse methods, such as conventional breeding, molecular breeding, and use of physical and chemical mutagens. Recently, soybean breeding has targeted the development of materials with enhanced nutritional qualities and functionality for industrial uses. In particular, enhanced availability of antioxidants, as an indicator of high nutritional and functional value, is an important breeding target desired in the food and cosmetics industries.

Metabolites that serve as antioxidants in soybean include phenolic compounds such as flavonoids (especially isoflavones) and anthocyanins [1,2]. In the most common soybean seed type with a yellowish-white seed coat, the predominant antioxidant compounds are isoflavones [3]. Isoflavones, a class of phytoestrogens, are effective not only as antioxidants but also as antiestrogens, and show antitumor activity and prevent cardiovascular disease [4,5,6]. Several legumes exhibit different health benefits due to their different phenolic composition [7]. For example, a higher content of hydroxybenzoic acids in lentils is effective in preventing cardiovascular problems [8]. Catechins and proanthocyanidins, which are abundantly present in red-colored lentils and dark-colored cranberry beans, have inhibition activity on low-density lipoprotein cholesterols as well as antioxidant activity [9].

Isoflavone content in the soybean seed depends on genotype and maturity rather than phenotypic traits, such as seed color and size [10,11]. Nevertheless, the antioxidant activity of soybean seeds can vary consistently with phenotypic traits. Typically, pigments in the seed coat are an important source of antioxidant activity. Black soybean seeds show higher antioxidant activity compared with that of yellow or green soybean seeds owing to the presence of anthocyanins, such as delphinidin and cyanidin glucosides, in the black seed coat [12]. Proanthocyanins, including epicatechin and procyanidin B2, are present in black and brown soybean seed coats that show antioxidant activity [13]. However, few accounts of the antioxidant activity and antioxidant compounds of brown soybean seeds have been reported. The selection of soybean cultivars with seed coats of a certain color and containing antioxidant compounds is important for the preservation of genetic diversity and to develop materials with new functional uses in industry.

With the development of novel breeding methods, the generation of increasingly diverse cultivars with high antioxidant potential has become feasible. In an effort to induce genetic mutations in plants with high levels of antioxidant properties, simple and efficient physical mutagens have been widely used. Gamma irradiation, as a physical mutagen, induces the production of reactive oxygen species and free radicals in plant cells, thereby providing antioxidant metabolites to stimulate the upregulation of antioxidant-associated genes and enzymes [14]. Increased antioxidant activity in γ-irradiated crops has been reported in diverse fruit and seed crops, such as rice, pistachio, and wheat as well as soybean [15,16,17]. Previous studies of soybean have reported that 100–200 Gy of γ-ray irradiation activates antioxidant responses, such as an increase in antioxidant enzyme activity, reduced glutathione content, and a decline in hydroxyl radical production [18]. The antioxidant activity of soybean seeds with a black, green, or yellow seed coats is increased by γ-ray irradiation [19]. Total flavonoid, phenolic compound, and isoflavone contents, in addition to antioxidant activity, are increased in γ-ray-treated soybean [20].

The use of physical mutagens in breeding enables the production of phenotypic diversity and fixation of a permanent high-antioxidant traits in a crop. Breeding mutant crops varying in genotypes and phenotypes can not only provide information as a breeding selection indicator, but can also assist in the generation of genetic resources and industrial materials through the evaluation of the antioxidant properties. Evaluation of the antioxidant functionality and contributors among diverse genotypes and phenotypes of soybean will provide valuable information for further development of the soybean industry.

Therefore, in the current study, we evaluated the antioxidant activity of soybean by investigating the radical-scavenging activities and reducing power of soybean “Danbaek” and six mutant lines differing in seed color induced by γ-ray irradiation of “Danbaek” seeds. We measured the contents of total phenolics and flavonoids and quantified individual anthocyanins, flavan-3-ols, and isoflavones by means of high-performance liquid chromatography (HPLC). To determine the location of antioxidant accumulation in response to γ-irradiation, seeds were separated into the seed coat and cotyledons (dehulled seeds), and their antioxidant properties were investigated. In addition, we investigated the antioxidant activity of 11 pure standard compounds of anthocyanins, flavan-3-ols, and isoflavones. Correlation analysis and principal component analysis of the association between antioxidant activity and the analyzed compounds were performed.

## 2. Materials and Methods

### 2.1. Chemicals

The standards for anthocyanins comprised delphinidin 3-*O*-glucoside chloride, petunidin 3-*O*-glucoside chloride, cyanidin chloride (Extrasynthese, Genay, France), and cyanidin 3-*O*-glucoside chloride (SinoStandards Bio-Tech Co., Ltd., Chengdu, China). The standards for flavan-3-ols consisted of catechin hydrate (Sigma Aldrich Co., St Louis, MO, USA) and epicatechin (Chemfaces, Wuhan, China). The standards for isoflavones comprised daidzein, glycitein, genistein, daidzin, glycitin, genistin (LC Laboratories, Woburn, MA, USA), acetyl daidzin, acetyl glycitin, acetyl genistin (Nacalai Tesque, Kyoto, Japan), malonyl daidzin, malonyl glycitin, and malonyl genistin (GenDEPOT, Katy, TX, USA).

### 2.2. Plant Materials

**Figure 1:**
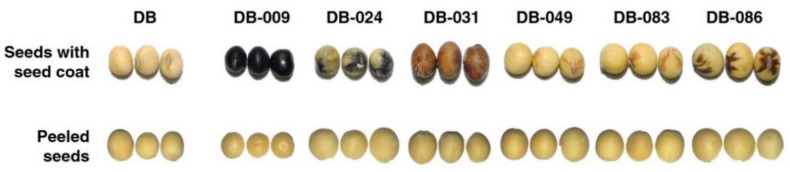
Seed color of *Glycine max* “Danbaek” (DB) and six mutant lines induced by irradiation with 250 Gy of γ-rays.

The mutant lines of soybean “Danbaek” (DB), a widely grown Korean soybean cultivar [21], were produced by the Korean Atomic Energy Research Institute (KAERI, Jeongeup, South Korea) in 2008 by irradiation with 250 Gy of γ-rays. In total, 1000 seeds of M1 DB mutants were sown in a research field of KAERI. Next, we generated individual mutants from M1 to M5 generations by a single-seed descent method and continued as bulks until the M12 generation (more schematic information described in Kim et al. [22]). Six genetically mutated seed lines that differed in seed coat color were selected. The seeds of DB and six mutant lines are shown in Figure 1. The seed coat was separated with tweezers after freeze drying.

### 2.3. Extraction for Measurement of Antioxidant Activity and Total Phenolic, Total Flavonoid, and Isoflavone Contents

The seed, seed coat, and dehulled seed (cotyledons) of each soybean line were ground using a commercial grinder. Ground samples (1 g) were extracted with 30 mL of 80% methanol (*v*/*v*). Extraction was performed by repeating the procedure three times over 24 h at 25 °C. The solvent was filtered through Whatman No. 2 filter paper (Whatman International Ltd., Maidstone, UK). Excess solvent was removed using a vacuum rotary evaporator (Eyela Co., Tokyo, Japan) at 40 °C. The extracts were lyophilized and stored at 4 °C.

### 2.4. Extraction for Determination of Anthocyanins and Flavan-3-ols

Extraction for the determination of anthocyanins and flavan-3-ols was performed in accordance with a previously described method with some modifications [23]. Briefly, each ground soybean seed, seed coat, and cotyledon sample (0.4 g) was extracted with 30 mL of acetone:water:formic acid (70:29.5:0.5, *v*/*v*/*v*) for 4 h at 25 °C in a shaking incubator. The extraction was performed by repeating the procedure four times. The solvent was then vacuum filtered through Whatman No. 2 filter paper (Whatman International Ltd.). The extraction solvent was evaporated using a vacuum rotary evaporator (Eyela Co.) at 40 °C. The concentrated extracts were lyophilized and redissolved in 50% methanol to a concentration of 30 mg mL^−1^ and stored in an amber tube at −20 °C until further analysis.

### 2.5. Antioxidant Activities

The 2,2-diphenyl-1-picrylhydrazyl (DPPH) and 2,2′-azino-bis (3-ethylbenzothiazoline-6-sulphonic acid) (ABTS) radical-scavenging activities of the extracts were determined using a previously described method [24]. For DPPH activity, 0.1 mM DPPH in 80% methanol (*v*/*v*) was adjusted 0.65 in absorbance value at 517 nm. Then, 0.05 mL of sample (1 mg mL^−1^) were added into 2.95 mL of DPPH solution. The absorbance was measured at 517 nm after a 30 min incubation at 23 °C in a dark chamber. For ABTS activity, 2.5 mM of ABTS and 1 mM of 2,2′-Azobis(2-amidinopropane) dihydrochloride in phosphate-buffered saline were mixed and heated at 70 °C for 40 min. The ABTS radical solution (0.98 mL) after filtering by a 0.45 μm syringe filter was added to 0.02 mL of sample (1 mg mL^−1^). The absorbance was measured at 734 nm after a 10 min incubation at 37 °C. DPPH and ABTS radical-scavenging activities were expressed as milligrams of vitamin C equivalents (VCE) per gram dry weight (DW).

The ferric-reducing ability of plasma (FRAP) assay was performed in accordance with the method of Benzie and Strain [25] with some modifications. The 300 mM acetate buffer (pH 3.6) was prepared by dissolving 3.1 g C_2_H_3_NaO_2_·3H_2_O and 16 mL acetic acid in 1 L distilled water. Solutions of 10 mM 2,4,6-tripyridyl-s-triazine (TPTZ) in 40 mM HCl and 20 mM FeCl_3_·6H_2_O in distilled water were prepared. The fresh FRAP solution was then prepared by mixing 25 mL acetate buffer, 2.5 mL TPTZ solution, and 2.5 mL FeCl_3_·6H_2_O just before use and heating to 37 °C. An aliquot of 950 µL FRAP solution was added to 50 µL extract and reacted for 30 min in the dark. The absorbance of the colored product (ferrous tripyridyltriazine complex) was measured at 593 nm. The FRAP activity was expressed as milligrams VCE per gram DW. Antioxidant activities of pure standard compounds were expressed as inhibition concentration 50 (IC_50_) values by evaluating the activities of a series of concentrations.

### 2.6. Total Phenolic and Flavonoid Contents

The total phenolic and total flavonoid contents were determined using previously published methods [26]. Total phenolics of soybean extracts were expressed as gallic acid equivalents (GAE) per gram DW. Total flavonoids of soybean extracts were expressed as catechin equivalents (CE) per gram DW.

### 2.7. Determination of Isoflavones Using Reversed-Phase HPLC

A sample (1 mg) of the 80% methanol extracts was diluted with 1 mL distilled water. The diluted extracts were filtered through a 0.45 µm hydrophilic PTFE syringe filter (Futecs Co., Ltd., Daejeon, Korea) and subjected to reversed-phase HPLC determination of isoflavones using a previously published method [27]. Briefly, the mobile phase consisted of (A) water with 0.1% formic acid and (B) acetonitrile with 0.1% formic acid. Gradient elution was performed as follows: 15–34% (B) over 0–60 min and then re-equilibrated to initial gradient. The flow rate was 1 mL min^−1^. Injection volume was 5 µL. Peaks were monitored at 254 nm.

### 2.8. Determination of Anthocyanins and Flavan-3-ols Using Reversed-Phase HPLC

Anthocyanins were analyzed using a Kinetex 5 μm C18 100A column (150 × 4.6 mm; Phenomenex, Torrance, CA, USA) in accordance with a previously described method with some modifications [28]. The flow rate was 0.8 mL min^−1^ by isocratic elution using water:methanol:formic acid (75:20:5). The injection volume was 10 µL and the column temperature was set at 27 °C. The eluents were monitored at 520 nm using a Waters 996 photodiode array detector (Waters Corporation, Milford, MA, USA). Four anthocyanin compounds, comprising delphinidin 3-*O*-glucoside, cyanidin 3-*O*-galactoside, cyanidin 3-*O*-glucoside, and petunidin 3-*O*-glucoside, were identified using standards. Peonidin 3-*O*-glucoside was identified on the basis of a previously published reference [29].

Flavan-3-ols were analyzed using an octadecylsilane column (Prontosil 120-5-C18-SH-EPS 5 μm (250 × 4.6 mm; Bischoff, Leonberg, Germany) in accordance with the method of Rios et al. [30] with some modifications. The flow rate of the mobile phase was 1.0 mL·min^−1^ and the injection volume was 10 μL. The linear gradient of the mobile phase consisted of solvents A (water:tetrahydrofuran:trifluoroacetic acid, 98:2:0.1, *v*/*v*/*v*) and B (acetonitrile). Separation was conducted by linear gradients of B into A as follows: starting with 13% B; 13–15% B, 0–5 min; 15–17% B, 5–20 min; 17–40% B, 20–24 min; 40–70% B, 24–27 min; 70% B for 3 min; and 13% B in 2 min. The eluents were monitored at 280 nm.

### 2.9. Statistical Analysis

All data were expressed as the mean ± standard error of three replicate experiments. All statistical analyses were performed using SAS software (Enterprise guide 7.1 version; SAS Institute Inc., Cary, NC, USA). Significant differences among the soybean lines in these experiments were evaluated using Tukey’s studentized range (honestly significant difference; HSD) test at the significance level of *p* < 0.05. Correlation analysis was performed by calculating Pearson’s correlation coefficients between the antioxidant activities and antioxidant compound groups with SAS software. Principal component analysis (PCA) score plots were generated on the basis of the antioxidant activities and antioxidant compound group data used for the correlation analysis with SAS software.

## 3. Results

### 3.1. Radical-Scavenging Activities and Reducing Power

**Figure 2:**
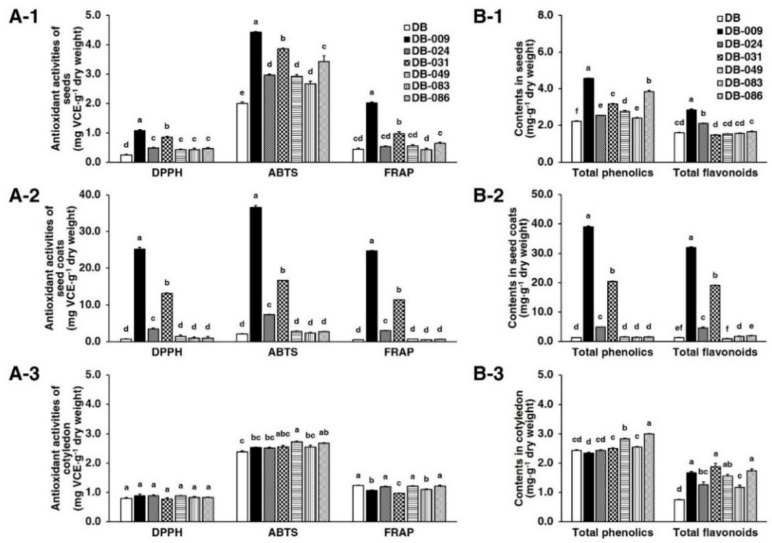
Antioxidant activities (**A**) and contents of total phenolics and total flavonoids (**B**) in the seed (A-1 and B-1), seed coat (A-2 and B-2), and cotyledons (A-3 and B-3) of *Glycine max* “Danbaek” and six γ-ray-irradiated mutant lines. Total phenolics and total flavonoids were expressed as mg gallic acid equivalents·g^−1^ dry weight and mg catechin equivalents·g^−1^ dry weight, respectively. Different letters above the bars indicate a significant difference at *p* < 0.05 using Tukey’s studentized range (HSD) test. VCE, vitamin C equivalents; DPPH, 2,2-diphenyl-1-picrylhydrazyl radical scavenging activity; ABTS, 2,2′-azino-bis (3-ethylbenzothiazoline-6-sulphonic acid radical scavenging activity; FRAP, ferric-reducing ability of plasma.

**Figure 3:**
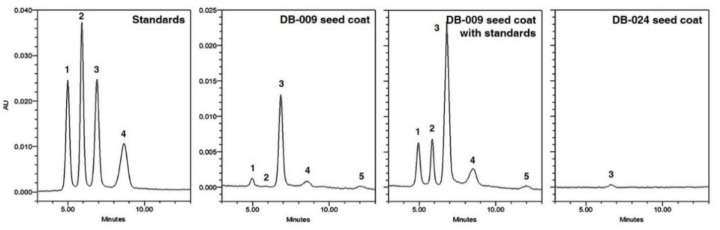
HPLC chromatograms for anthocyanins in the seed coat of the DB-009 and DB-024 γ-ray-induced mutant lines of *Glycine max* “Danbaek”. 1, Delphinidin 3-*O*-glucoside; 2, cyanidin 3-*O*-galactoside; 3, cyanidin 3-*O*-glucoside; 4, petunidin 3-*O*-glucoside; 5, peonidin 3-*O*-glucoside.

Antioxidant activities in the seed, seed coat, and cotyledons of DB and the six mutant lines were evaluated by determining radical-scavenging activities (DPPH and ABTS) and reducing power using the FRAP assay (Figure 2A). With regard to antioxidant activities in the seed (Figure 2A-1), all six mutant lines selected for different seed coat colors (Figure 1) showed higher DPPH and ABTS radical-scavenging activities than those of DB. In particular, DB-009, which produced a uniformly black seed coat, showed the highest radical-scavenging activity in all three assays, exhibiting 1.07 mg VCE·g^−1^ DW for DPPH, 4.43 mg VCE·g^−1^ DW for ABTS, and 2.01 mg VCE·g^−1^ DW for FRAP. DB-031, which produced a uniformly brown seed coat, showed the second-highest activities, exhibiting 0.85 mg VCE·g^−1^ DW for DPPH, 3.86 mg VCE·g^−1^ DW for ABTS, and 0.97 mg VCE·g^−1^ DW for FRAP. DB-086 was the third-highest ranked line only in the ABTS assay, expressing 3.44 mg VCE·g^−1^ DW. The other three mutant lines did not show distinct differences in antioxidant activities. With regard to the seed coat (Figure 2A-2), DB-009 showed the highest antioxidant activity, exhibiting 25.23 mg VCE·g^−1^ DW for DPPH, 36.54 mg VCE·g^−1^ DW for ABTS, and 24.76 mg VCE·g^−1^ DW for FRAP. DB-031 showed approximately 50% of the antioxidant activity detected for DB-009 but activity was still significantly higher in comparison with those of the other mutant lines. DB-024, a mutant line with a partially black seed coat, was third-highest ranked for antioxidant activity. The remainder of the lines showed similarly low antioxidant activities. In the cotyledons (Figure 2A-3), no lines showed a significant difference in antioxidant activity in the DPPH assay. Slight differences in antioxidant activities were detected in the ABTS and FRAP assays among the mutant lines compared with activities in the seed and seed coat, but in no instance was the difference remarkable.

### 3.2. Total Phenolics and Total Flavonoids

The contents of total phenolics (TP) and total flavonoids (TF) in the seed, cotyledons, and seed coat were determined (Figure 2B). The TP in the seed was higher in all six mutant lines compared with that of DB (Figure 2B-1). DB-009 showed the highest TP content (4.57 mg GAE·g^−1^ DW), followed by DB-086 and DB-031. The black-seeded DB-009 and partially black-seeded DB-024 showed higher TF contents in the seed than that of DB, whereas the other lines showed similar seed TF contents to that of DB. In the seed coat (Figure 2B-2), the TP and TF contents showed significant differences among the lines, with the content ranging from 1.30 to 39.05 mg GAE·g^−1^ DW for TP and from 0.98 to 31.98 mg CE·g^−1^ DW for TF. The seed coats of DB-009, DB-031, and DB-024 showed higher TP contents (39.05, 20.45, and 4.91 mg GAE·g^−1^ DW, respectively) than that of DB. These three lines also showed higher TF contents with a similar pattern to that observed for TP content. Less variation in TP content in the cotyledons was observed among the lines, which ranged from 2.34 to 3.00 mg GAE·g^−1^ DW (Figure 2B-3). The TF contents in the cotyledons of DB-009, DB-031, and DB-086 were higher than those observed for the other lines. However, the TF contents were low in all lines (<2 mg CE·g^−1^ DW).

### 3.3. Anthocyanins

Anthocyanins are an important class of plant pigments and antioxidants in soybeans with a black seed coat [31]. The anthocyanin composition of DB and the mutant lines was analyzed (Table 1). In the seed, cyanidin 3-*O*-glucoside was detected only in DB-009. Five anthocyanins, comprising delphinidin 3-*O*-glucoside, cyanidin 3-*O*-galactoside, cyanidin 3-*O*-glucoside, petunidin 3-*O*-glucoside, and peonidin 3-*O*-glucoside, were detected in the seed coat of the black-seeded line DB-009 (Figure 3). Cyanidin 3-*O*-glucoside was the predominant anthocyanin (28.39 mg·100 g^−1^ DW) detected in the seed coat of DB-009 and was the only anthocyanin detected in DB-024 (a partially black seed coat line; 0.39 mg·100 g^−1^ DW). Anthocyanins were not detected in the cotyledons of any lines. In addition, none of the anthocyanins analyzed were detected in any other lines except for DB-009 and DB-024, even in the seed coat.

### 3.4. Flavan-3-ols

The contents of catechin and epicatechin, which are types of flavan-3-ols, were analyzed in the soybean lines (Table 2). In the seed, catechin content did not differ significantly among the lines, whereas epicatechin content was higher in DB-031 (15.30 mg·100 g^−1^ DW) and DB-009 (5.72 mg·100 g^−1^ DW) than that of the other lines (<0.7 mg·100 g^−1^ DW). In the seed coat, epicatechin content showed a broad variation among the lines, ranging from 0 to 193.29 mg·100 g^−1^ DW, with the highest content detected in DB-031. The DB-009 seed coat (black in color) showed a high content of epicatechin (68.70 mg·100 g^−1^ DW). Epicatechin in yellow-colored seed coats was detected only in trace amounts. In the cotyledons, catechin and epicatechin contents did not show significant differences among the lines and were lower than those detected in the seed coat.

### 3.5. Isoflavones

Isoflavones, which show various physiological bioactivities, including antioxidant activity, are an important group of flavonoids in soybean. The contents of the predominant isoflavones in soybean, including aglycones (daidzein, glycitein, and genistein), β-glucosides (daidzin, glycitin, and genistin), acetyl glucosides (acetyl daidzin, acetyl glycitin, and acetyl genistin), and malonyl glucosides (malonyl daidzin, malonyl glycitin, and malonyl genistin), were analyzed in the present study (Table 3).

According to previous studies, isoflavones in soybean are present predominantly in the cotyledons rather than the seed coat, and in the form of malonyl glycosides [2]. The present results were consistent with these observations for all six γ-ray-induced lines. Isoflavone accumulation, especially of malonyl glucosides, was enhanced in the seed of the mutant lines (Table 3). Seeds of DB-086 showed the highest content of total isoflavones among the mutant lines with an increase of 6.9 times compared with that of DB seeds. Seeds of DB-009, which showed the highest antioxidant activity, contained the lowest isoflavones content among the mutant lines (168.76 mg·100 g^−1^ DW). The content of isoflavones in the seed showed strong similarity to the content in the cotyledons, whereas the isoflavone content in the seed coat of some γ-ray-induced mutant lines showed different patterns. The seed coats of DB-024 and DB-031 showed the highest total isoflavone content and included a high proportion of malonyl glucosides. In contrast, the DB-009 seed coat showed the lowest total isoflavone content among the mutant lines, but contained the highest aglycone content.

### 3.6. Correlation Analysis and PCA

Analysis of correlations between antioxidant activities and compounds revealed which compound groups predominantly possessed antioxidant potential in each part of the seed (Table 4). The TP, TF, total anthocyanin (TA), and total flavan-3-ol (TF 3-ols) contents showed a strong positive and significant correlation with DPPH, ABTS, and FRAP activities in the seed and the seed coat. No significant correlation was observed between total isoflavone (TI) content and antioxidant activities in the seed and the seed coat. However, TI showed a significant correlation with ABTS activity in the cotyledons (*r* = 0.753, *p* < 0.001). The ABTS activity of the cotyledons also showed a strong correlation with TP, whereas DPPH and FRAP activities of the cotyledons were not positively correlated with any antioxidant compound group.

**Figure 4:**
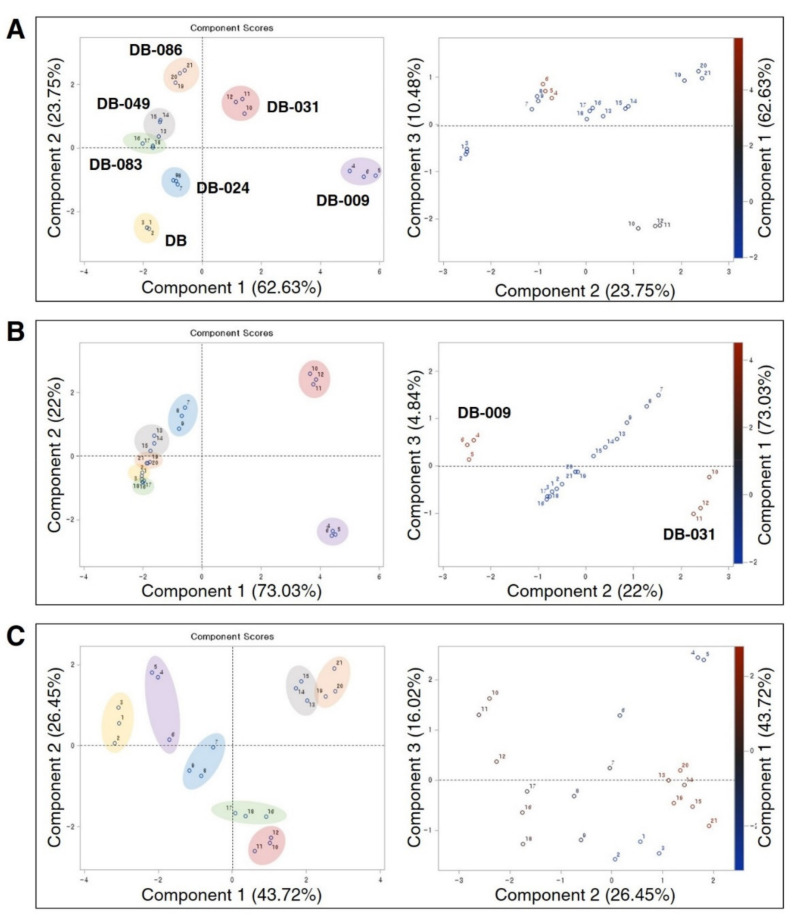
Principal component analysis score plots generated from antioxidant activities and compound data used for correlation analysis for the seed (**A**), seed coat (**B**), and cotyledons (**C**) of *Glycine max* “Danbaek” and six γ-ray-induced mutant lines. The numbers in the plots represent the scores for DB (1–3), DB-009 (4–6), DB-024 (7–9), DB-031 (10–12), DB-049 (13–15), DB-083 (16–18), and DB-086 (19–21).

The PCA score plots provided an overview of the similarities and differences in antioxidant activities and compounds among the six γ-ray-induced mutant lines in the seed, seed coat, and cotyledons. The black-coated DB-009 and the brown-coated DB-031 were segregated from the other soybean lines by component 1 in the seed (62.63%) and seed coat (73.03%) (Figure 4A,B). The lines DB-009 and DB-031 were separated by components 2 and 3 for both the seed and the seed coat. With regard to the seed coat, all lines except DB-009 and DB-031 were tightly grouped (Figure 4B). The PCA scores for the cotyledons were widely distributed among the lines relative to those for the seed and seed coat with 43.72% of component 1 (Figure 4C).

### 3.7. Antioxidant Activities of Pure Standard Compounds

The antioxidant activities of 11 pure standard compounds, comprising anthocyanins (delphinidin 3-*O*-glucoside, cyanidin 3-*O*-glucoside, and petunidin 3-*O*-glucoside), flavan-3-ols (catechin and epicatechin), and isoflavones (genistein, genistin, acetyl genistin, and malonyl genistin), were evaluated (Table 5). The antioxidant activity of anthocyanin (delphinidin 3-*O*-glucoside, cyanidin 3-*O*-glucoside, petunidin 3-*O*-glucoside, and cyanidin) and flavan-3-ol (catechin and epicatechin) standards was significantly higher than that of isoflavones.

Anthocyanins and flavan-3-ols showed similar DPPH activities, with IC_50_ values ranging from 165.56 to 213.95 µg mL^−1^ and 151.90 to 167.88 µg mL^−1^, respectively. Isoflavones showed little DPPH activity, even at a high concentration of 10 mg mL^−1^. The ABTS activity was slightly higher for flavan-3-ols (IC_50_ 43.76 and 49.26 µg mL^−1^) than that of anthocyanins (IC_50_ 64.6–79.07 µg mL^−1^). Genistein, an aglycone isoflavone, showed higher ABTS activity (IC_50_ of 31.15 µg mL^−1^) than those of anthocyanin and flavan-3-ol compounds. The glycosides of genistein showed the lowest ABTS activity compared with all other compounds. The FRAP activity was slightly higher for anthocyanins (IC_50_ 49.87–57.94 µg mL^−1^) than for flavan-3-ols (IC_50_ 70.95 and 76.17 µg mL^−1^). The FRAP activity of isoflavones ranged from 14% to 43%, even at a high concentration of 2.5 mg mL^−1^.

## 4. Discussion

Soybean seeds of different colors vary in chemical composition and show differences in antioxidant efficiency [1,12,29,32]. Darker-colored soybean seeds show the highest antioxidant potential [1,12,32]. In particular, the high antioxidant activity of black soybean seeds and representative anthocyanin antioxidants in the seed have been widely reported [2,28,31,33]. In accordance with the seed coat pigmentation, we selected six lines with black, partially black, brown, partially brown, or yellowish-white seed coats raised from seeds mutated by γ-ray irradiation. Only black-pigmented seeds (the uniformly black-pigmented DB-009 and partially black-pigmented DB-024 lines) contained anthocyanins in the seed coat. These results were consistent with previous studies that detected five anthocyanins, of which cyanidin 3-*O*-glucoside was the pigment primarily responsible for the black color of the seed coat [29,33]. Consistent with a previous study [33], anthocyanins in the seed coat significantly contributed to the antioxidant activity of the black-seeded mutant line. Although anthocyanins are known to be the predominant antioxidants in black-seeded soybean, the present results showed that catechin and epicatechin were detected in large amounts and also strongly contributed to the high antioxidant activity of black-seeded soybean.

All mutant lines with brown or yellow seeds lacked anthocyanins in all seed parts. The seed and seed coat of brown-seeded DB-031 showed the second-highest antioxidant activity after the black-seeded line DB-009, despite the absence of anthocyanins in seeds of the former line. Compared with the frequent studies on antioxidants in black and yellow soybean seeds, brown-seeded soybeans have rarely been evaluated for antioxidant activity and associated compounds. In the present study, the high antioxidant activity of brown seeds was attributable to the high catechin and epicatechin contents in the seed coat. These results were supported by the correlation analysis and assessment of the antioxidant activity of standard compounds (Table 4 and Table 5). Catechin and epicatechin, which exhibit a white to light brown color, show antioxidant activity in many plant species, such as cocoa and green tea [34,35]. Takahata et al. [36] reported that the antioxidant activity of brown soybean seeds is due to proanthocyanidin, an oligomer of catechin and epicatechin, and that proanthocyanidin in black and brown soybean seeds is mostly present in a polymerized form. The brown color of wild type Arabidopsis testa (seed coat) is due to the oxidation of proanthocyanin distributed to its precursors mainly in the endothelium layer [37]. The antioxidant enzyme activity of proanthocyanin-deficient mutant Arabidopsis seeds is over-activated compared to wild type [38]. The absence of proanthocyanin upregulation an antioxidant system of seeds implies that proanthocyanin is a putative antioxidant in response to stress circumstance. According to Kovinich et al. [13], brown soybean seeds contain high contents of epicatechin and epicatechin-based soluble procyanidins. The present results indicate that epicatechin in brown seed coats is potentially the main antioxidant contributor in brown soybean seeds. The line DB-086, with partially darker brown seeds, showed low contents of flavan-3-ols in the seed coat and cotyledons. Condensed tannins formed by condensation of flavans can contribute to a darker brown pigmentation [36,39]. The presence of isoflavones with an iron-chelating moiety bound to the cell wall pectin in the soybean seed coat may also induce a dark red-brown coloration [40].

Isoflavone, which is known to be an important phenolic compound in soybean, was not associated with seed color in the present study, as previously reported [11,41]. In addition, the TI content in the seed and seed coat was not correlated with antioxidant activity. According to Kumar et al. [32], the isoflavone content is similar in yellow, green, and black soybean seeds and is not associated with DPPH and FRAP antioxidant activities. With regard to the present results on DPPH, ABTS, and FRAP activities, TI content showed a positive correlation only with ABTS activity in the cotyledons (*r* = 0.753, *p* < 0.001). This result indicated that the high ABTS activity in the seed and cotyledons of DB-086 was attributable to the high isoflavone content. The TI content of any seed part, including the cotyledons, was not significantly correlated with DPPH and FRAP activities, which was consistent with the antioxidant activities of the isoflavone standards (Table 5). In particular, DPPH activity was barely detected, even at a high concentration of the isoflavone standards. A previous study reported that isoflavones are only active in the ABTS assay because the assay shows a stronger and faster response to antioxidants [42]. In addition, ABTS radicals are chemically stable within a wider pH range (pH 1–8) than DPPH radicals (pH 4–8) [43]. Therefore, antioxidant assays should be selectively applied depending on the plant materials and chemicals under assessment.

In the ABTS and FRAP assays with isoflavone standards, the aglycone genistein showed the highest antioxidant activity and malonyl genistin showed the lowest activity. Given that soybean seeds contain higher quantities of malonyl glycosides than aglycones, as shown in the present results and previous reports [2,41], isoflavones were not considered to contribute notably to antioxidant activity. In the cotyledons, TI content was strongly correlated with TP and TF contents. Thus, the positive correlation of TP and TF contents with ABTS activity in the cotyledons results from the strong positive correlation with TI content. The antioxidant activity of anthocyanin and flavan-3-ol standards was significantly higher than that of isoflavone standards (Table 5). Similarly, it was previously reported that anthocyanins and flavan-3-ols show high ABTS and FRAP activities, and that monomeric flavan-3-ols show higher antioxidant activity than polymeric flavan-3-ols [44]. Given the high antioxidant activity of anthocyanins and monomeric flavan-3-ols, the contribution of isoflavones to the antioxidant activity in soybean seeds was not remarkable, although isoflavones in the cotyledons contributed significantly to ABTS activity.

The seeds contained substantially larger amounts of isoflavones (90.84–623.75 mg·100 g^−1^ DW) than anthocyanins (0–17.06 mg·100 g^−1^ DW) or flavan-3-ols (3.24–18.19 mg·100 g^−1^ DW). Nevertheless, the antioxidant activity of seeds showed a strong positive correlation with anthocyanins and flavan-3-ols and no correlation with isoflavones (Table 4). These results indicated that the antioxidant activity of soybean seeds is more strongly dependent on anthocyanins and flavan-3-ols than on isoflavones. Furthermore, the cotyledons show a significantly lower content of low-molecular-weight phenolic compounds with high antioxidant activity than that of the seed coats [2]. The antioxidant activity of pigmented seeds is contributed by the seed coat, as evidenced by the high concentration of anthocyanins or flavan-3-ols in the seed coat. In addition to anthocyanins and flavan-3-ols, the seed coat of black soybean seeds contains more varied and abundant phenolic compounds than the cotyledons [45]. Various components in seeds can induce diversity of health benefits. Anthocyanin has been widely reported for its strong antioxidant effect. On the other hand, high catechin and epicatechin in hop seeds had not only an antioxidant effect but also antimicrobial activity [46]. In addition, isoflavone aglycones in soybean seeds have been widely studied and proved as having an antiestrogen effect rather than antioxidant activity [4]. Simple phenolics such as vanillic acid and 3,4-dihydroxybenzoic acid are abundant in lentil seed coats and have beneficial effects against intestinal inflammation [47,48]. In this study, it is suggested that flavan-3-ols are considered as an obvious indicator to determine antioxidant activity in soybean seed coat colors.

In the PCA score plots for the seed and seed coat, DB-009 and DB-031 were distinguished from the other mutant lines by component 1, owing to their high antioxidant activity and high flavan-3-ol content. In addition, the scatterplots of components 2 and 3 for the seed and seed coat showed clear separation of DB-009 and DB-031. In the scatterplot for the seed coat, DB-009 and DB-031 were distantly separated, but in different vector directions, from the tightly grouped remaining lines. Thus, it can be concluded that the antioxidative factors of DB-009 and DB-031 differ. Although flavan-3-ols were present in the seed coat of black and brown soybean seeds, the absence of anthocyanins in the seed coat of brown seeds would account for this result.

## 5. Conclusions

This study provides information on the antioxidant components, and their localization and accumulation, among six soybean lines differing in seed color that were mutated from one cultivar. The mutant lines were characterized as showing high anthocyanins content (DB-009, black seed coat), high epicatechin content (DB-031, brown seed coat), or high isoflavone content (DB-086, yellowish-white seed coat). On the basis of the results of correlation analysis and PCA of the antioxidant activities and components of the mutant soybean lines, the crucial antioxidant compounds were revealed to be anthocyanins in black seeds and flavan-3-ols in brown and black seeds. Cyanidin 3-*O*-glucoside was the dominant antioxidant compound in black seed coats and comprised ~84% of the total anthocyanin content in the seed coat and 100% in the cotyledons. A high content of epicatechin, a type of flavan-3-ol, reflects the high antioxidant activity of brown seed coats. An increase in the antioxidant activity of soybean seeds is dependent on the contents of anthocyanins and flavan-3-ols in the seed coat rather than isoflavone content in the cotyledons. The present results provide information that is especially useful for applications of soybean in the food and cosmetics industries, and in breeding for soybean seed color as a valuable antioxidant resource.

## Figures and Tables

**Table 1:** Anthocyanin content (mg·100 g^−1^ dry weight) in the seed, seed coat, and cotyledons of γ-ray-induced mutant lines of *Glycine max* “Danbaek”.

		Delphinidin 3-*O*-Glucoside	Cyanidin 3-*O*-Galactoside	Cyanidin 3-*O*-Glucoside	Petunidin 3-*O*-Glucoside	Peonidin 3-*O*-Glucoside ^z^	Total Anthocyanin
DB-009	Seed	n.d.^y^	n.d.	17.06 ± 2.02	n.d.	n.d.	17.06 ± 2.02
	Seed coat	2.13 ± 0.15	0.18 ± 0.03	28.39 ± 0.58	2.41 ± 0.16	1.41 ± 0.09	33.71 ± 0.80
	Cotyledon	n.d.	n.d.	n.d.	n.d.	n.d.	n.d.
							
DB-024	Seed	n.d.	n.d.	n.d.	n.d.	n.d.	n.d.
	Seed coat	n.d.	n.d.	0.39 ± 0.06	n.d.	n.d.	0.39 ± 0.06
	Cotyledon	n.d.	n.d.	n.d.	n.d.	n.d.	n.d.

^z^ Peonidin 3-*O*-glucoside content is expressed as mg cyanidin 3-*O*-glucoside equivalents·100 g^−1^ dry weight. ^y^ n.d., compound not detected.

**Table 2:** Catechin and epicatechin contents (mg·100 g^−1^ dry weight) in the seed, seed coat, and cotyledons of *Glycine max* “Danbaek” and six γ-ray-induced mutant lines.

Parts	Lines	(+)-Catechin	(-)-Epicatechin	Total Flavan-3-ols
Seed	DB	4.37 ± 0.15 ab	0.56 ± 0.10 cd	4.93 ± 0.09 c
	DB-009	4.94 ± 0.10 a	5.72 ± 0.08 b	10.66 ± 0.03 b
	DB-024	3.16 ± 0.38 c	0.21 ± 0.03 d	3.37 ± 0.41 d
	DB-031	2.90 ± 0.21 c	15.30 ± 0.16 a	18.19 ± 0.16 a
	DB-049	3.67 ± 0.10 bc	0.70 ± 0.01 c	4.36 ± 0.06 c
	DB-083	3.02 ± 0.14 c	0.21 ± 0.14 d	3.24 ± 0.01 d
	DB-086	4.53 ± 0.06 ab	0.63 ± 0.09 c	5.16 ± 0.09 c
Seed coat	DB	0.00 d	0.00 c	0 ± 0 c
	DB-009	8.26 ± 0.42 b	68.70 ± 6.16 b	76.96 ± 6.01 b
	DB-024	5.43 ± 0.21 bc	9.65 ± 0.66 c	15.08 ± 0.47 c
	DB-031	13.23 ± 2.23 a	193.29 ± 6.86 a	206.52 ± 7.94 a
	DB-049	2.72 ± 0.32 cd	1.64 ± 0.85 c	4.37 ± 1.02 c
	DB-083	2.33 ± 0.59 cd	0.00 c	2.33 ± 0.59 c
	DB-086	2.64 ± 0.62 cd	0.00 c	2.64 ± 0.62 c
				
Cotyledon	DB	5.03 ± 0.16 b	0.64 ± 0.19 a	5.67 ± 0.35 ab
	DB-009	6.91 ± 0.55 a	0.00 a	6.91 ± 0.55 a
	DB-024	3.49 ± 0.02 cd	0.33 ± 0.27 a	3.82 ± 0.18 cd
	DB-031	2.37 ± 0.23 d	0.39 ± 0.21 a	2.76 ± 0.21 d
	DB-049	4.38 ± 0.31 bc	0.71 ± 0.11 a	5.09 ± 0.29 bc
	DB-083	2.30 ± 0.07 d	0.27 ± 0.15 a	2.56 ± 0.11 d
	DB-086	4.95 ± 0.27 b	0.37 ± 0.16 a	5.31 ± 0.24 b

Different superscript letters among the soybean lines for each organ and compound indicate a significant difference at *p* < 0.05 using Tukey’s studentized range (HSD) test.

**Table 3:** Isoflavones (mg·100g^−1^ dry weight) in the seed, seed coat, and cotyledons of *Glycine max* “Danbaek” and six γ-ray-induced mutant lines.

		Total Aglycones	Total β-Glucosides	Total Acetyl Glucosides	Total Malonyl Glucosides	Total Isoflavones
Seed	DB	1.99 ± 0.10 b	31.24 ± 1.35 d	0.94 ± 0.18 a	56.66 ± 1.73 f	90.84 ± 2.97 f
	DB-009	9.13 ± 2.38 b	36.68 ± 2.56 d	1.90 ± 0.72 a	121.06 ± 2.17 e	168.76 ± 1.88 e
	DB-024	6.74 ± 1.40 b	65.41 ± 9.25 cd	4.84 ± 2.18 a	186.93 ± 12.39 d	263.93 ± 6.61 d
	DB-031	7.75 ± 2.11 b	100.41 ± 12.82 ab	7.18 ± 3.30 a	280.35 ± 26.93 c	395.69 ± 18.94 c
	DB-049	8.69 ± 1.11 b	94.03 ± 0.50 bc	8.65 ± 4.33 a	352.83 ± 16.75 b	464.19 ± 22.32 b
	DB-083	8.37 ± 1.22 b	98.45 ± 9.59 abc	5.50 ± 2.78 a	301.12 ± 5.87 bc	413.44 ± 11.17 bc
	DB-086	18.25 ± 2.70 a	129.10 ± 1.38 a	14.40 ± 7.20 a	462.00 ± 4.67 a	623.75 ± 9.72 a
Seed coat	DB	0.60 ± 0.20 b	9.73 ± 0.68 de	0.24 ± 0.16 b	44.28 ± 3.12 de	54.85 ± 3.71 cd
	DB-009	30.59 ± 5.18 a	1.80 ± 0.58 f	4.82 ± 2.34 a	12.43 ± 2.51 e	49.64 ± 5.29 cd
	DB-024	1.61 ± 0.69 b	29.37 ± 1.28 a	0.50 ± 0.21 ab	151.05 ± 12.04 a	182.53 ± 12.92 a
	DB-031	3.09 ± 1.03 b	25.05 ± 0.82 b	1.06 ± 0.75 ab	132.22 ± 11.48 ab	161.42 ± 12.27 a
	DB-049	0.51 ± 0.05 b	20.90 ± 0.42 c	0.41 ± 0.06 ab	99.65 ± 8.30 bc	121.47 ± 8.69 b
	DB-083	0.55 ± 0.19 b	6.99 ± 0.46 e	0.33 ± 0.05 ab	33.74 ± 0.76 e	41.61 ± 1.16 d
	DB-086	0.58 ± 0.01 b	11.78 ± 0.70 d	0.27 ± 0.07 b	69.29 ± 1.22 cd	81.92 ± 0.63 c
Cotyledon	DB	0.68 ± 0.17 bc	26.39 ± 2.71 d	1.33 ± 0.22 a	89.14 ± 2.64 f	117.54 ± 4.25 e
	DB-009	0.48 ± 0.02 c	24.22 ± 0.92 d	0.95 ± 0.03 a	123.34 ± 7.72 e	148.98 ± 7.19 e
	DB-024	0.99 ± 0.12 bc	40.13 ± 4.61 cd	1.66 ± 0.20 a	177.25 ± 7.31 d	220.04 ± 11.86 d
	DB-031	1.34 ± 0.16 ab	53.94 ± 3.49 bc	0.98 ± 0.09 a	235.95 ± 2.05 c	292.21 ± 5.47 c
	DB-049	0.98 ± 0.17 bc	68.53 ± 4.02 ab	1.18 ± 0.17 a	280.35 ± 2.87 b	351.04 ± 5.25 b
	DB-083	0.64 ± 0.05 c	56.39 ± 3.86 bc	1.09 ± 0.19 a	240.74 ± 1.51 c	298.86 ± 5.21 c
	DB-086	1.93 ± 0.20 a	80.69 ± 7.13 a	1.73 ± 0.19 a	318.69 ± 13.99 a	403.04 ± 20.77 a

Different superscript letters among the soybean lines for each organ and compound indicate a significant difference at *p* < 0.05 using Tukey’s studentized range (HSD) test.

**Table 4:** Correlation coefficients between antioxidant activities and metabolite groups in the seed, seed coat, and cotyledons of *Glycine max* “Danbaek” and six γ-ray-induced mutant lines.

		DPPH	ABTS	FRAP	Total Phenolics	Total Flavonoids	Total Anthocyanins (A)	Total Flavan-3-ols (B)	Total Isoflavones (C)	A + B	A + B + C
Seed	DPPH	1									
	ABTS	0.907 ***	1								
	FRAP	0.913 ***	0.840 ***	1							
	Total phenolics	0.739 ***	0.881 ***	0.849 ***	1						
	Total flavonoids	0.643 **	0.579 **	0.804 ***	0.653 **	1					
	Total anthocyanins (A)	0.765 ***	0.670 **	0.929 ***	0.758 ***	0.889 ***	1				
	Total flavan-3-ols (B)	0.740 ***	0.655 **	0.553 *	0.429 ^ns^	0.080 ^ns^	0.278 ^ns^	1			
	Total isoflavones (C)	−0.176 ^ns^	0.154 ^ns^	−0.301 ^ns^	0.139 ^ns^	−0.470 *	−0.418 ^ns^	−0.057 ^ns^	1		
	A + B	0.940 ***	0.827 ***	0.945 ***	0.759 ***	0.649 **	0.837 ***	0.758 ***	−0.316 ^ns^	1	
	A + B + C	−0.128 ^ns^	0.201 ^ns^	−0.256 ^ns^	0.182 ^ns^	−0.443 *	−0.380 ^ns^	−0.018 ^ns^	0.998 ***	−0.268 ^ns^	1
Seed coat	DPPH	1									
	ABTS	0.946 ***	1								
	FRAP	0.997 ***	0.936 ***	1							
	Total phenolics	0.987 ***	0.982 ***	0.983 ***	1						
	Total flavonoids	0.990 ***	0.974 ***	0.988 ***	0.999 ***	1					
	Total anthocyanins (A)	0.738 ***	0.911 ***	0.714 ***	0.829 ***	0.807 ***	1				
	Total flavan-3-ols (B)	0.790 ***	0.556 **	0.810 ***	0.700 ***	0.724 ***	0.189 ^ns^	1			
	Total isoflavones (C)	0.100 ^ns^	−0.080 ^ns^	0.125 ^ns^	−0.012 ^ns^	−0.001 ^ns^	−0.370 ^ns^	0.375 ^ns^	1		
	A + B	0.876 ***	0.680 ***	0.891 ***	0.804 ***	0.824***	0.344 ^ns^	0.987 ***	0.298 ^ns^	1	
	A + B + C	0.679 ***	0.445 *	0.703 ***	0.570 **	0.590 **	0.055 ^ns^	0.900 ***	0.730 **	0.870 ***	1
Cotyledon	DPPH	1									
	ABTS	0.083 ^ns^	1								
	FRAP	0.116 ^ns^	0.092 ^ns^	1							
	Total phenolics	0.023 ^ns^	0.747 ***	0.405 ^ns^	1						
	Total flavonoids	0.148 ^ns^	0.549 *	−0.560 **	0.333 ^ns^	1					
	Total anthocyanins (A)	-	-	-	-	-	-				
	Total flavan-3-ols (B)	0.274 ^ns^	0.001 ^ns^	0.339 ^ns^	0.041 ^ns^	−0.020 ^ns^	-	1			
	Total isoflavones (C)	−0.024 ^ns^	0.753 ***	0.046 ^ns^	0.850 ***	0.532 *	-	−0.346 ^ns^	1		
	A + B	0.274 ^ns^	0.001 ^ns^	0.339 ^ns^	0.041 ^ns^	−0.020 ^ns^	-	1.000 ***	−0.346 ^ns^	1	
	A + B + C	−0.019 ^ns^	0.757 ***	0.051 ^ns^	0.855 ***	0.534 *	-	−0.332 ^ns^	0.999 ***	−0.332 ^ns^	1

**Table 5:** Antioxidant activities of 11 pure standard compounds.

Class of Compounds	Compounds	DPPH Scavenging Activity IC_50_ (µg mL^−1^)	ABTS Scavenging Activity IC_50_ (µg mL^−1^)	FRAP Activity EC_50_ (µg mL^−1^)
Anthocyanins	Delphinidin 3-*O*-glucoside	165.56	73.16	49.87
	Cyanidin 3-*O*-glucoside	192.20	64.62	51.96
	Petunidin 3-*O*-glucoside	213.95	79.07	57.94
Flavan-3-ols	Catechin	167.88	49.26	76.17
	Epicatechin	151.90	43.76	70.95
		Inhibition at 10 mg mL^−1^		Effectiveness at 2.5 mg mL^−1^
Isoflavones	Genistein	<10%	31.15	43.78%
	Genistin	<10%	191.92	23.24%
	Acetyl genistin	<10%	192.27	20.68%
	Malonyl genistin	<10%	258.39	14.17%

## Data Availability

Not applicable.
